# Long-term sickness absence in a working population: development and validation of a risk prediction model in a large Dutch prospective cohort

**DOI:** 10.1186/s12889-020-08843-x

**Published:** 2020-05-15

**Authors:** Lennart R. A. van der Burg, Sander M. J. van Kuijk, Marieke M. ter Wee, Martijn W. Heymans, Angelique E. de Rijk, Goedele A. Geuskens, Ramon P. G. Ottenheijm, Geert-Jan Dinant, Annelies Boonen

**Affiliations:** 1 Department of Family Medicine, Care and Public Health Research Institute (CAPHRI), Maastricht University, Maastricht, The Netherlands; 2 Department of Internal Medicine, Division of Rheumatology, Maastricht University Medical Centre and Care and Public Health Research Institute (CAPHRI), Maastricht University, Maastricht, The Netherlands; 3 Department of Clinical Epidemiology and Medical Technology Assessment, Maastricht University Medical Centre, Maastricht, The Netherlands; 4 Department of Epidemiology and Biostatistics, Amsterdam UMC, Vrije Universiteit Amsterdam, Amsterdam Public Health, Amsterdam, The Netherlands; 5 Department of Social Medicine, Care and Public Health Research Institute (CAPHRI), Maastricht University, Maastricht, The Netherlands; 6 Netherlands Organisation for Applied Scientific Research TNO, Leiden, The Netherlands

**Keywords:** Prediction model, Prediction, Long-term sickness absence, Prospective cohort study, Prevention, Calibration, Discrimination, Development, External validation, Working persons

## Abstract

**Background:**

Societal expenditures on work-disability benefits is high in most Western countries. As a precursor of long-term work restrictions, long-term sickness absence (LTSA) is under continuous attention of policy makers. Different healthcare professionals can play a role in identification of persons at risk of LTSA but are not well trained. A risk prediction model can support risk stratification to initiate preventative interventions. Unfortunately, current models lack generalizability or do not include a comprehensive set of potential predictors for LTSA. This study is set out to develop and validate a multivariable risk prediction model for LTSA in the coming year in a working population aged 45–64 years.

**Methods:**

Data from 11,221 working persons included in the prospective Study on Transitions in Employment, Ability and Motivation (STREAM) conducted in the Netherlands were used to develop a multivariable risk prediction model for LTSA lasting ≥28 accumulated working days in the coming year. Missing data were imputed using multiple imputation. A full statistical model including 27 pre-selected predictors was reduced to a practical model using backward stepwise elimination in a logistic regression analysis across all imputed datasets. Predictive performance of the final model was evaluated using the Area Under the Curve (AUC), calibration plots and the Hosmer-Lemeshow (H&L) test. External validation was performed in a second cohort of 5604 newly recruited working persons.

**Results:**

Eleven variables in the final model predicted LTSA: older age, female gender, lower level of education, poor self-rated physical health, low weekly physical activity, high self-rated physical job load, knowledge and skills not matching the job, high number of major life events in the previous year, poor self-rated work ability, high number of sickness absence days in the previous year and being self-employed. The model showed good discrimination (AUC 0.76 (interquartile range 0.75–0.76)) and good calibration in the external validation cohort (H&L test: *p* = 0.41).

**Conclusions:**

This multivariable risk prediction model distinguishes well between older workers with high- and low-risk for LTSA in the coming year. Being easy to administer, it can support healthcare professionals in determining which persons should be targeted for tailored preventative interventions.

## Background

Participation in paid work is one of the most important social roles of individuals in our society. Paid work is a source of income, protects against social exclusion and gives meaning to life [1–3]. Moreover, restrictions in work participation (e.g. sickness absence or permanent work disability) cause a substantial burden on societal expenditures in most Organisation for Economic Co-operation and Development (OECD) countries [4]. Worker productivity is a continuum, ranging from normal productivity over presenteeism and prolonged sick leave to withdrawal from paid work. It is recognized that return to work is unlikely when individuals have become work disabled [5, 6]. Early recognition and prevention of long-term restrictions in work participation, e.g. long-term sickness absence (LTSA) (usually defined as more than 4–6 weeks of sickness absence), have therefore become an important target in several countries, including the Netherlands [7]. Most healthcare professionals, such as general practitioners who are usually first consulted when a (medical) problem arises, are not well trained in identifying individuals at risk for restrictions in work participation. Risk prediction models could support early identification by those healthcare professionals, and ensure timely initiation of targeted interventions to prevent long-term work restrictions [6, 8, 9].

There are few studies that have published multifactorial risk prediction models that identify working persons at risk of LTSA [10]. However, these models frequently address selected professional groups or work sectors and not a broader general working population [11–15]. Other limitations of those studies were that the risk prediction model was not externally validated [16], was mostly developed in Scandinavian cohorts and likely not generalizable to other countries, or did not include a comprehensive set of variables relevant for sustainable work participation in their available dataset for prediction model development [16–18].

The Study on TRansitions in Employment, Ability and Motivation (STREAM) conducted in the Netherlands is suitable for development and external validation of a prediction model for LTSA in a general working population aged 45–64 years. The strength of STREAM is that a wide variety of variables important for sustainable work participation were collected that are not currently included in most occupational health surveys. Therefore, this study set out to develop and externally validate an easy-to-assess multivariable risk prediction model for LTSA in the coming year in a general working population aged 45–64 years.

## Methods

### Study design and participants

STREAM is an ongoing prospective cohort among persons aged 45 to 64 years in the Netherlands stratified by age and employment status. From the inception of the cohort in 2010 (T1) onwards, participants completed an online questionnaire on topics such as work characteristics, health, employment status and transitions, work ability, and work productivity. A more detailed description of the STREAM study design has been published previously [19]. At the time of the fifth measurement of the STREAM cohort (2015), a second cohort of participants was recruited, consisting of persons aged 45–49 years and employed persons in the other age groups (50–54, 55–59, 60–64).

For development of the current risk prediction model, data from the first (T1, 2010) and the second (T2, 2011) measurement were used. This cohort comprised 15,118 persons who participated in STREAM of which 82.2% responded at T2 (*N* = 12,430). Subjects were included in the analyses if they were employed at time of inclusion (including self-employment), but were excluded if they received a fulltime disability pension, or had been on LTSA in the previous year (see definition below).

For external validation, data from the second cohort recruited at the time of the scheduled fifth measurement (T5, 2015) and 1 year follow-up (T6, 2016) were used. This cohort consisted of 6728 persons at T5 and 77.4% responded at T6 (*N* = 5218).

### Definition of long-term sickness absence

LTSA in the year of follow up was defined as ≥28 accumulated working days and was assessed through self-report in the follow-up survey.

### Predictor variables of long-term sickness absence

#### Available predictors

The online questionnaire included a wide variety of variables covering fourteen different domains: demographic characteristics (e.g. age), health and well-being (e.g. perceived health), work-related factors (e.g. working conditions), knowledge and skills (e.g. developmental proactivity), social factors (e.g. work-family balance), financial factors (e.g. household income), motivation to work (e.g. job satisfaction), ability to work (e.g. Work Ability Index (WAI)), opportunity to work (e.g. support by colleagues), productivity at work (e.g. presenteeism), employment status and transitions, mastery (i.e. Pearlin Mastery Scale), job intentions (e.g. to stop working) and coping styles (i.e. Utrecht Coping List). A more detailed description of all (sub) domains and questionnaires included in STREAM has been published previously [19]. Most of the (sub) domains have been shown in previous publications to be important for sustainable employability and cover the different components of the International Classification of Functioning, Disability, and Health (ICF) [1].

#### Selection of candidate predictors for the model

Variables available at T1 were included as candidate predictors if they had been inquired among working participants, including self-employed participants. This resulted in 141 candidate predictors from 11 different domains. Several steps were undertaken to further reduce the amount of candidate predictors to be included in the full statistical model. First, a literature search was performed to identify previously published prediction models and individual predictors specific for LTSA [11–13, 16–18, 20]. These predictors were recorded and similar or identical variables in STREAM were identified. Thereafter, an expert consensus group meeting including all authors listed above was organized to reach consensus which variables available in STREAM should be included in the full statistical model. As a guiding principle, it was agreed that (at least) one candidate predictor from each applicable domain should be included in the full statistical model. To inform the expert consensus group, members had insight into all univariate associations between the predictor variables and the outcome as well as results from the literature search (data not shown). If two or more predictors within a domain showed overlap in content or were statistically highly correlated, the most feasible predictor for a clinical setting was selected. For example, the self-rated SF-12 Mental Component Score (MCS) and Center for Epidemiologic Studies Depression scale (CES-D-10) showed great overlap in content, an equally strong association with the outcome and a strong statistical correlation between them. The MCS was eventually chosen because it requires less items to complete (6 vs. 10 items). Finally, it was pre-specified to maintain the variables age, gender and level of education in the model because these predictors were frequently included in previously published models (face validity). This expert meeting resulted in 27 candidate predictors across 11 domains that were included in the full statistical model (description in Additional file 1).

### Statistical analysis

Missing data in the development and the validation cohort were imputed using multiple imputation techniques according to the method described by Van Buuren et al. [21] We created m = 30 imputed datasets and used predictive mean matching for imputing continuous predictors, polytomous regression for categorical predictors and logistic regression for dichotomous predictors. All 27 candidate predictors and the outcome LTSA were included in the imputation model.

A logistic regression analysis was performed to estimate regression coefficients of the association between predictors and LTSA during the coming year. First, the ‘full statistical model’ was computed including all 27 candidate predictors in each of the imputed datasets. We then used backward stepwise elimination across all imputed datasets using *p* > 0.05 as a rule to remove predictors from the model to create a smaller and therefore more practical final prediction model.

The performance of the final prediction model was evaluated using the Area Under the Receiver Operating Characteristic (ROC) Curve (AUC), which reflects how well the model discriminates between those persons with and without LTSA in the coming year. The AUC has a range from 0.5 (i.e. no discriminative ability) to 1.0 (perfect discriminative ability). Calibration of the final prediction model, i.e. the correspondence of the predicted and observed probabilities, was evaluated using calibration plots and the Hosmer-Lemeshow goodness of fit test. To assess the degree of overfitting we used bootstrapping techniques for internal validation with 1000 bootstrap samples in each of the imputed datasets. The estimated shrinkage factor was applied to the regression coefficients to arrive at the final model.

Sensitivity analyses included: 1) complete case analyses (i.e. participants with no missing values on any of the predictor variables or outcome), for both model development and validation, and 2) stratified analyses for employed and self-employed participants. To externally validate the final prediction model, we assessed its predictive performance and calibration in the validation cohort. All analyses were performed with R version 3.4.4. in RStudio 1.1.442 (RStudio Inc., Boston, MA) using the following packages: *mice*, *rms* and *psfmi*.

## Results

**Fig. 1:**
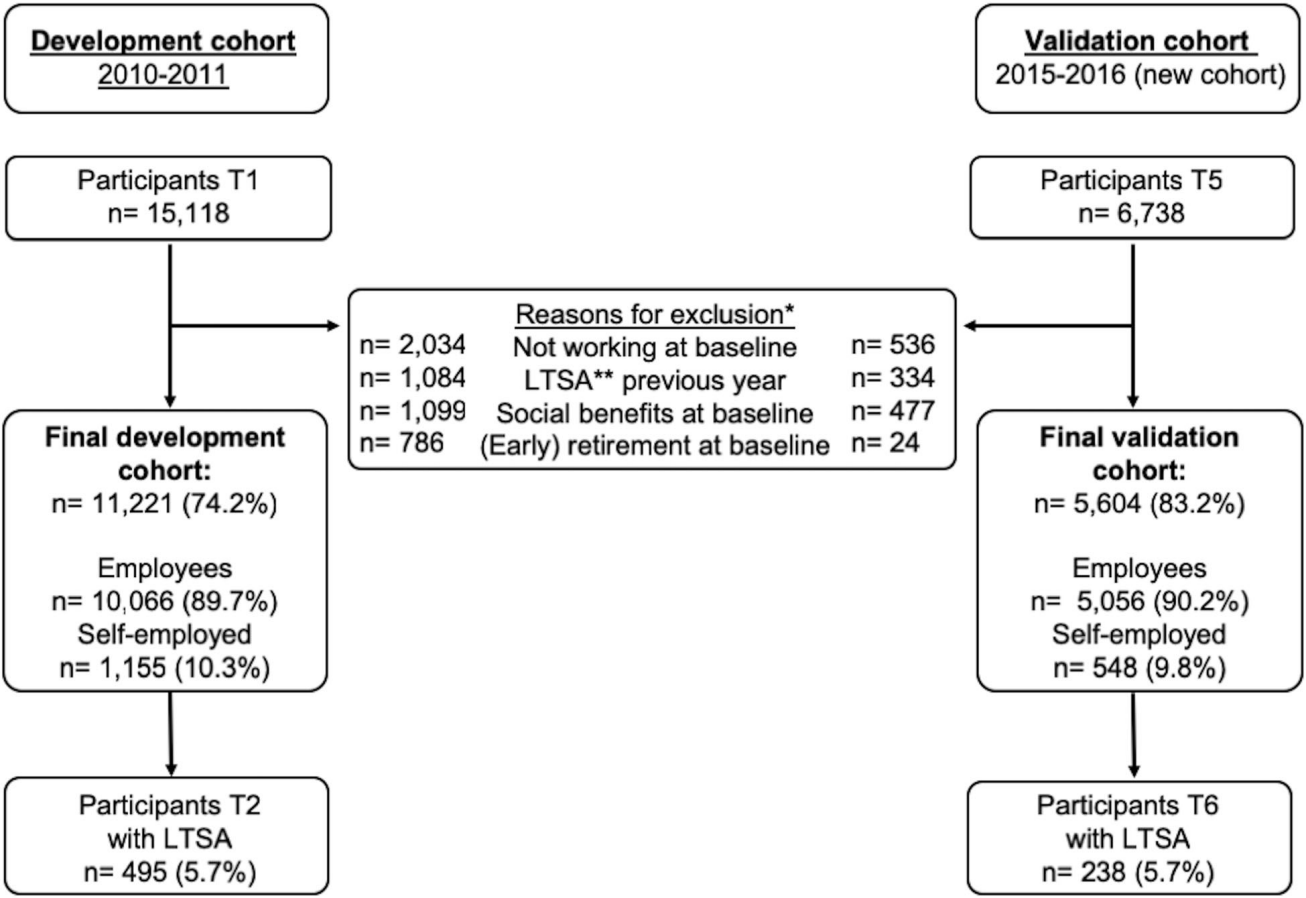
Flow chart of the development and validation cohort used for the analyses in this study. *More than one reason can apply to one participant. **Long-term sickness absence (LTSA) was defined as ≥28 accumulated work days of sick leave during 1 year of follow-up

After exclusion of participants currently not working, receiving a fulltime disability pension or with LTSA in the previous year, 11,221 of the 15,118 participants at T1 (74.2%) were included in the development cohort. Similarly, 5604 of the 6728 participants at T5 (83.3%) were included in the validation cohort (see Fig. 1). Participants in the validation cohort were on average younger, less frequently male, and reported a somewhat better physical health (see Table 1). After 1 year of follow-up, 495 participants (5.7%) in the development cohort and 238 participants (5.7%) in the validation cohort reported LTSA.

**Table 1:** Baseline characteristics of participants in the development and validation cohort

	**Level**	**Development cohort**	**Validation cohort**	
		***n*** **= 11,221**	***n*** **= 5604**	***p*-value**
Female, (%)		4819 (42.9)	2753 (49.1)	< 0.001
Age, mean (SD)		53.9 (5.4)	50.2 (5.2)	< 0.001
Educational level^a^, n(%)	Low	2924 (26.1)	1390 (24.8)	0.12
	Medium	4349 (38.8)	2253 (40.2)	
	High	3948 (35.2)	1961 (35.0)	
SF-12 physical health^b^, mean (SD)		52.4 (7.0)	52.2 (7.4)	0.07
Physically fit^c^, n(%)		4502 (40.4)	2535 (45.4)	< 0.001
Physical job load^d^, mean (SD)		1.8 (0.9)	1.9 (0.9)	< 0.001
Knowledge and skills match the job, n(%)	Bad/mediocre	488 (4.4)	255 (4.6)	0.54
	Reasonable/good	10,687 (95.6)	5323 (95.4)	
Major life events previous year, n(%)	0	5877 (52.4)	3239 (57.8)	< 0.001
	1	3577 (31.9)	1640 (29.3)	
	≥2	1767 (15.7)	725 (12.9)	
Work ability^e^, mean (SD)		8.0 (1.4)	8.1 (1.4)	0.10
Sickness absence days previous year, mean (SD)		2.9 (5.1)	2.7 (4.9)	0.01
Employment status, n(%)	Employee	10,066 (89.7)	5056 (90.2)	0.30
	Self-employed	1155 (10.3)	548 (9.8)	

SD = standard deviation, SF = Short Form Health Survey

^a^Low: lower general secondary educational, preparatory secondary vocational education. Medium: intermediate vocational training, higher general secondary education, pre-university education. High: higher vocational education, university education

^b^Weighted summary score (range 0–100) assessing physical health using 6 items of the 12-Item Short-Form Health Survey. Higher scores indicating better perceived health

^c^Intensive physical exercise at least ≥3 days per week for ≥20 min

^d^Average of five items (range: 1 = never, 5 = always) from the Dutch Musculoskeletal Questionnaire [22]

^e^Measured with the first item of the Work Ability Index (WAI) [23]

Numbers of missing values were low for all candidate predictor variables (less than 3% for each variable, see Additional file 1). Follow-up data for the outcome variable were missing from 2540 of the 11,221 participants (22.6%) in the development cohort and from 1432 of the 5604 participants (25.6%) in the validation cohort. To examine possible selective loss to follow-up, we compared baseline characteristics of participants missing at the follow-up measurement with participants that were not missing. Table 3 in Additional file 1 shows some statistically significant differences which are not clinically relevant. All candidate predictors and the outcome in both the development and validation cohort were imputed using the methods described above.

### Development of the prediction model

Eleven of the 27 predictors were retained in the final prediction model: age (continuous), gender (male vs. female), education (low vs. medium; low vs. high), self-rated physical health (SF-12 Physical Component Score (PCS)), quartiles of increasing good health, lowest/poorest quartile is reference), being physically fit (at least 3 days of intensive physical exercise for more than 20 min or more, no vs. yes), amount of physical job load (average of 6 items, lowest three quartiles combined vs. highest quartile), knowledge and skills matching the job (bad/mediocre vs. reasonable/good), self-rated work ability (first item of the WAI, good (score 8–10) vs. average (6/7), good vs. poor (0–5)), the number of sickness absence days in the previous year (none vs. 1–5 days, none vs. 6–10 days, none vs. 11–27 days), being self-employed (no vs. yes) and the number of major life events in the previous year (none vs. 1 event, none vs. 2 or more events). Good self-rated physical health (odds ratio (OR) 0.46 (95%-confidence interval (CI) 0.36–0.59), lowest quartile vs. highest quartile), with poor self-rated work ability (OR 4.70 (95%-CI 3.50–6.30), good vs. poor) and a high number of sickness absence days in the previous year (OR 4.68 (95%-CI 3.77–5.80), none vs. 11–27 days) showed the strongest univariate associations with LTSA in the development cohort (see Table 2). Sensitivity analyses using the complete cases resulted in the same predictor variables retained in the final prediction model and similar univariate associations with LTSA (see Additional file 1, table 4). We found similar results for employed and self-employed persons and therefore decided to develop one prediction model that included both groups and with self-employment as one of the predictor variables (data not shown).

**Table 2:** Univariate associations and multivariable regression coefficients of the predictors in the final model of the development cohort

		**Univariate**	**Multivariable**	
**Predictor**	**Level**	**OR (95%-CI)**^**a**^	**OR**	**Coefficient**^**b**^
Female gender		1.26 (1.05–1.51)	1.10	0.09
Age, per year		1.02 (1.00–1.03)	1.00	0.007
Educational level^c^ (ref: low)	Medium	1.00 (0.84–1.19)	0.88	−0.13
	High	0.75 (0.62–0.91)	0.83	−0.19
SF-12 physical health^d^ (ref: 1st quartile, poorest health)	2nd quartile	0.97 (0.80–1.18)	0.55	−0.59
	3rd quartile	0.51 (0.41–0.65)	0.42	−0.87
	4th quartile	0.46 (0.36–0.59)	0.41	−0.90
Physically fit^e^		0.77 (0.64–0.94)	0.80	−0.22
Physical job load^f^ (ref: 1st-3rd quartile, less demanding)	4th quartile	1.63 (1.35–1.98)	1.33	0.29
Knowledge and skills match the job (ref: bad/mediocre)	Reasonable/good	0.43 (0.30–0.59)	0.62	−0.48
Major life events previous year (ref: none)	1	1.10 (0.92–1.33)	1.12	0.11
	≥2	1.62 (1.32–2.00)	1.43	0.35
Work ability^g^ (ref: good)	Average	1.40 (1.14–1.71)	1.10	0.10
	Poor	4.70 (3.50–6.30)	2.28	0.82
Sickness absence days previous year (ref: none)	1–5	0.90 (0.73–1.12)	1.39	0.33
	6–10	2.25 (1.76–2.87)	2.53	0.93
	11–27	4.68 (3.77–5.80)	3.84	1.35
Self-employed		0.49 (0.33–0.73)	0.57	−0.57
*Intercept*				−2.55

^a^Pooled Odds Ratio (95% confidence interval) from the m = 30 multiple imputed datasets

^b^Pooled regression coefficients and intercept from the m = 30 multiple imputed datasets. An individuals predicted probability can be computed using the logistic regression formula P (LTSA) = 1/(1 + exp.(−LP), in which ‘exp’ denotes e-raised-to-the-power-of. The LP is the linear predictor, i.e. the linear sum of all predictor values multiplied by their regression coefficients, or − 2.55 + 0.09*gender (female = 1) + 0.007*age (years) -0.13*education (medium education = 1) -0.19*education (high education = 1) -0.59*physical health (2nd quartile = 1) -0.87*physical health (3rd quartile = 1) -0.90*physical health (4th quartile = 1) -0.22*physically fit (yes = 1) + 0.29*physical job load (4th quartile = 1) -0.48*knowledge (reasonable/good = 1) + 0.11*major life events (one event = 1) + 0.35*major life events (two or more = 1) + 0.10*work ability (average = 1) + 0.82*work ability (poor = 1) + 0.33*sickness absence (1–5 days = 1) + 0.93*sickness absence (6–10 days = 1) + 1.35*sickness absence (11–27 days = 1) -0.57*employment status (self-employed = 1)

^c^Low: lower general secondary educational, preparatory secondary vocational education. Medium: intermediate vocational training, higher general secondary education, pre-university education. High: higher vocational education, university education

^d^Weighted summary score (range 0–100) assessing physical health using 6 items of the 12-Item Short-Form Health Survey. Higher scores indicating better perceived physical health. 1st quartile < 46.1, 2nd quartile = 46.1–54.1, 3rd quartile = 54.2–56.5, 4th quartile > = 56.6

^e^Intensive physical exercise ≥3 days per week for ≥20 min

^f^Average of five items (range: 1 = never, 5 = always) from the Dutch Musculoskeletal Questionnaire [22]. 1st-3rd quartile < 2.4, 4th quartile > = 2.4

^g^Measured with the first item of the Work Ability Index (WAI) [23]. Good = 8–10, average = 6/7, poor = 0–5

### Validation of the prediction model

**Fig. 2:**
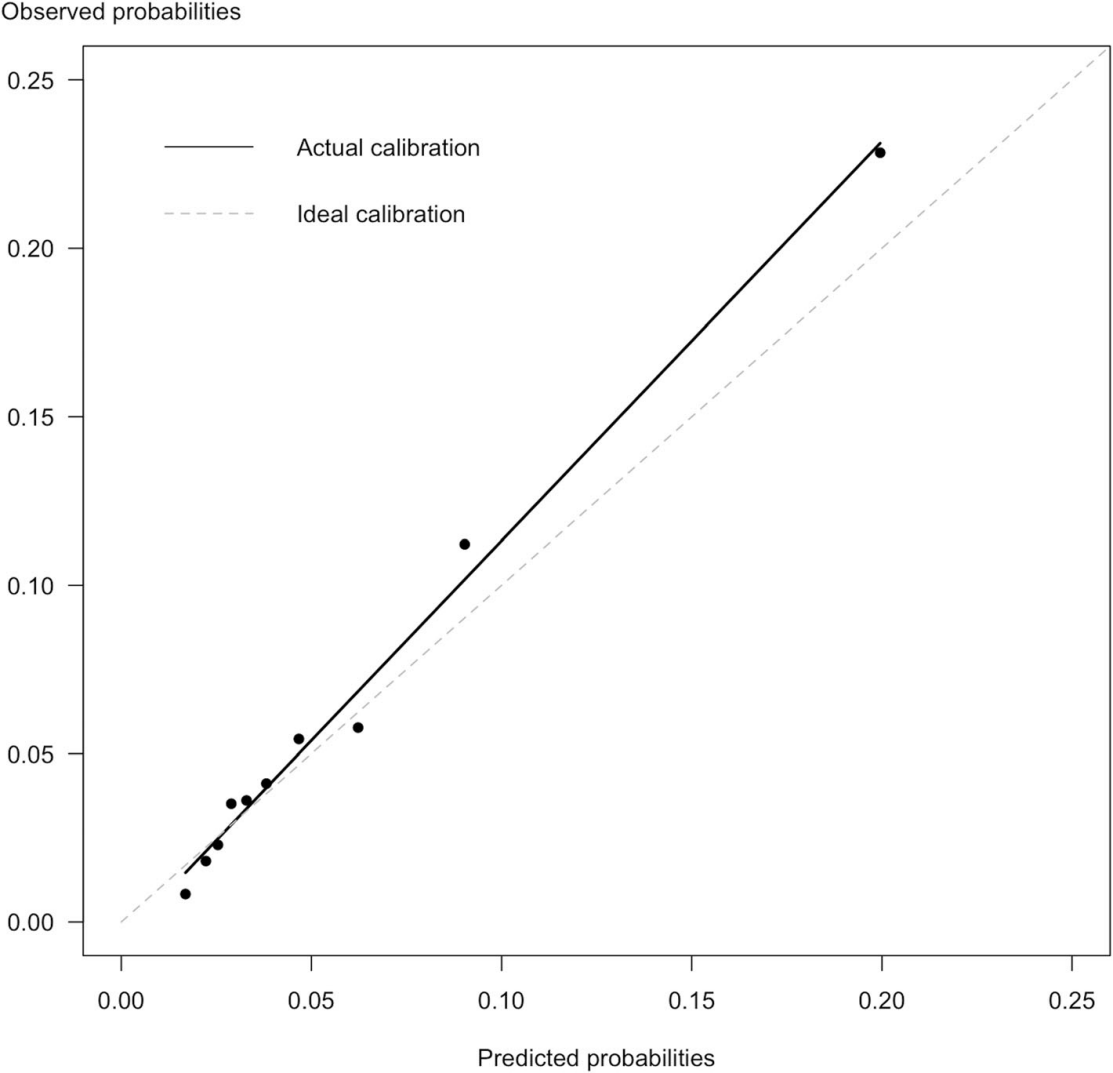
Calibration plot visualizing the mean predicted LTSA by the model against observed frequencies per decile of predicted risk in the validation cohort. Hosmer-Lemeshow test: *p* = 0.41

Internal validation of the prediction model using bootstrapping across m = 30 imputed datasets showed good discriminative ability: pooled median AUC 0.73 (interquartile range (IQR) 0.73–0.74) for predicting LTSA in the coming year. In the validation cohort, discrimination remained similar as observed in the development cohort: pooled median AUC 0.76 (IQR 0.75–0.76). The prediction model showed good calibration (predicted LTSA risks by the model plotted against the observed LTSA frequencies) in the validation cohort (see Fig. 2, H&L test: *p* = 0.41). No further updating of the prediction model was necessary. Sensitivity analyses using the complete cases in the validation cohort yielded similar results (see Additional file 1, table 4 and Fig. 1).

## Discussion

We developed and externally validated a model to predict LTSA of 28 or more work days in the coming year in a general working population aged 45–64 years. The following eleven easy to assess predictors were retained in the final multivariable model: older age, female gender, lower level of education, poor self-rated physical health, low weekly physical activity, high self-rated physical job load, knowledge and skills not matching the job, high number of major life events in the previous year, poor self-rated work ability, high number of sickness absence days in the previous year and not being self-employed. The prediction model discriminates well between working persons with and without LTSA and showed good calibration in the external validation cohort.

Previously published prediction models, mostly developed within routinely collected occupational health datasets, found that low physical health, prior (long-term) sickness absences and having mental health issues are important predictors of future LTSA [11, 13, 16, 17]. However, mental health was not a predictor of LTSA in our model, possibly due to some collinearity with other variables in the full multivariable model (e.g. major life events, emotional job demands) or due to the constitution of the cohort which included only older working persons and diverse professional sectors and different contract types. Also, mental health issues may be more important in certain working populations or sectors (e.g. younger persons, white collar workers) but this should be confirmed in future studies updating this prediction model. Our model confirms the role of previous sickness absence and self-reported physical health in predicting future LTSA. It is of note that the level of perceived limitations in physical functioning predict future LTSA better than the health condition possibly underlying these limitations, such as musculoskeletal disease or multi-morbidity, which were eliminated from the full multivariable model that included self-reported physical health and physical job load. Predictors assessing social and financial factors, emotional job demands and autonomy were eliminated from the full multivariable model, although some of these have been shown to be predictors of LTSA in a few previous studies [11, 16, 17]. Possibly the broader professional background but also older age of this cohort might account for these differences. We found that other factors are important for predicting LTSA which were not included in previous studies. Knowledge and skills matching the current job, the number of major life events in the previous year, and not being self-employment proved to be strong predictors of future LTSA. We found no important effect modification by employment status (employee vs. self-employed) and therefore decided to develop one prediction model that included both groups. However, not being self-employed was an important predictor for future LTSA and was therefore included in our final multivariable model.

One of the strengths of the present study is that the STREAM cohort includes a number of new candidate predictors for LTSA not previously addressed in occupational health surveys. Secondly, this large and prospective population-based study provides sufficient statistical power to develop and validate an accurate prediction model. Another strength was that the feasibility of candidate predictors, i.e. how easily can the information be collected by a healthcare professional, was regarded as important to ensure future practical purposes. All methods used for model development and validation in this study are in accordance with the Transparent Reporting of a multivariable prediction model for Individual Prognosis Or Diagnosis (TRIPOD) statement [24].

One limitation of this study was that loss to follow-up was present in both the development and validation cohort which could have introduced attrition bias. However, the participants that were lost to follow-up did not differ importantly from participants that were not lost to follow-up, we used multiple imputation techniques to deal with the missing values and sensitivity analysis on complete cases yielded similar results. The impact of the social security system or the working culture within a nation on the risk of sickness absence and disability pension has been shown to be different in the Netherlands compared to other European countries [25–27]. This prediction model was developed and validated in a cohort of working persons aged 45 to 64 years and therefore potentially not generalizable to younger age-groups because other factors may also be relevant in younger working persons (e.g. family-work balance, job security) [28]. It is possible that this prediction model needs to be adjusted (e.g. adjusting regression coefficients/intercept, adding or removing predictors) for use in other countries and transferability can be an interesting topic for future research.

### Practical implications and directions for future research

This prediction model was developed to support healthcare professionals, such as general practitioners, public health workers and occupational specialists, in identifying working persons of 45 years or older at high risk of LTSA in the coming year. After identification, supportive interventions should be considered, ranging from raising awareness or providing simple advice for modifying lifestyle or working conditions, referral to a medical or occupational specialist, or a preventative rehabilitation program in case of a complex problem. Given the low prevalence of LTSA in the working population, and thus the risk for a high false-positive rate, it is essential to have a simple screening tool and low cost interventions for the persons at risk [10]. The impact of the proposed prediction model on clinical decision making and short- and long-term patient outcome and cost-effectiveness should be studied in a (cluster) randomized design. Before such a study is designed some facilitators and barriers to further improve the ease of use of this prediction model should be taken into account [29]. Firstly, automated calculations (e.g. in a web-based format or integration in the electronic patient records) will ease the use for healthcare professionals. Secondly, an optimal cut-off point needs to be determined (i.e. balance between the harm of a false-positive classification and the benefit of a true-positive classification) while also taking into account the availability of resources after referral of high-risk persons. Finally, our model has not only the promise to target persons at risk, but could also serve to identify new and potentially modifiable risk factors at the level of companies or workplaces (e.g. physical fitness) [10, 30].

## Conclusions

We developed and validated a prediction model for long-term sickness absence in the coming year that showed good discrimination and calibration in a general population of working persons aged 45–64 years in the Netherlands. Future studies should investigate the transferability of this prediction model to other settings, age-groups and countries as well as the effects on clinical decision making and patient outcome.

## Supplementary information

**Additional file 1.** ONLINE SUPPLEMENTARY INFORMATION. Online Supplementary Table 1, Online Supplementary Table 2, Online Supplementary Table 3, Online Supplementary Table 4, Online Supplementary Figure 1.

## Data Availability

The data that support the findings of this study are available from TNO Healthy Living (Leiden, the Netherlands) but restrictions apply to the availability of these data, which were used under license for the current study, and so are not publicly available. Data are however available from the authors upon reasonable request and with permission of TNO Healthy Living (Leiden, the Netherlands).

## Abbreviations

AUC: Area Under the Curve
CES-D-10: Center for Epidemiologic Studies Depression scale
CI: Confidence Interval
H&L: Hosmer-Lemeshow test
ICF: International Classification of Functioning, Disability, and Health
IQR: InterQuartile Range
MCS: Mental Component Score of the 12-item Short Form Health Survey
OR: Odds Ratio
PCS: Physical Component Score of the 12-item Short Form Health Survey
ROC: Receiver Operating Characteristic
SD: Standard Deviation
SF-12: 12-item Short Form Health Survey
STREAM: Study on Transitions in Employment, Ability and Motivation
LTSA: Long-Term Sickness Absence
WAI: Work Ability Index
