# Supplementary material for: Long-term sickness absence in a working population: development and validation of a risk prediction model in a large Dutch prospective cohort
Source: BMC Public Health. 2020 May 15;20:699. doi: 10.1186/s12889-020-08843-x (PMC7227258; doi:10.1186/s12889-020-08843-x)
Supplement: Supplementary file 1 — Additional file 1. ONLINE SUPPLEMENTARY INFORMATION. Online Supplementary Table 1, Online Supplementary Table 2, Online Supplementary Table 3, Online Supplementary Table 4, Online Supplementary Figure 1. [file 12889_2020_8843_MOESM1_ESM.pdf]

ONLINE SUPPLEMENTARY INFORMATION

**Online Supplementary Table 1. Description of the 27 candidate predictors included in the 'full statistical model'**		
**Domain**	**Description of predictor**	**Instrument**
Demographics	Gender	-
Demographics	Age	-
Demographics	Educational level	-
Health & well-being	Physical health	SF-12 (Short Form Health Survey) Physical Component Score (PCS)
Health & well-being	Physical activity	-
Health & well-being	Mental health	SF-12 (Short Form Health Survey) Mental Component Score (MCS)
Health & well-being	Vitality	SF-36 (Medical Outcomes Study 36-item Short-Form Health Survey)
Health & well-being	Body mass index	-
Health & well-being	Diseases at baseline	Netherlands Working Conditions Survey (NWCS)
Work-related factors	Physical job load	Dutch Musculoskeletal Questionnaire
Work-related factors	Supervisory role	Netherlands Working Conditions Survey (NWCS)
Work-related factors	Autonomy	Job Content Questionnaire (JCQ)
Work-related factors	Job demands	Job Content Questionnaire (JCQ)
Work-related factors	Emotional job demands	Copenhagen Psychosocial Questionnaire (COPSOQ)
Knowledge & skills	Knowledge and skills match the job	-
Social factors	Major life events previous year	-
Financial factors	Financial situation household	-
Work ability	Work ability	Work Ability Index (WAI)
Productivity	Sickness absence previous year	Netherlands Working Conditions Survey (NWCS)
Employment status & transitions	Self-employment	-
Motivation	Interesting at work	Netherlands Working Conditions Survey (NWCS)
Motivation	Independence at work	Netherlands Working Conditions Survey (NWCS)
Motivation	Atmosphere at work	Netherlands Working Conditions Survey (NWCS)
Motivation	Good salary	Netherlands Working Conditions Survey (NWCS)
Motivation	Ability to change work hours at work	Netherlands Working Conditions Survey (NWCS)
Motivation	Job satisfaction	Netherlands Working Conditions Survey (NWCS)
Other concepts	Mastery skills	Pearlin Mastery Scale (PMS)

Online Supplementary Table 2. Number of missingness for all candidate predictors in the development and validation cohort		
**Variable**	**Development**	**Validation**
	n=11,221	n=5,604
Gender	0	0
Age	0	1 (0.02)
Educational level	0	0
SF-12 physical health	318 (2.83)	189 (3.37)
Physical activity	83 (0.74)	24 (0.43)
Physical job load	31 (0.28)	6 (0.11)
Knowledge and skills match the job	46 (0.41)	25 (0.46)
Major life events previous year	0	0
Work ability	27 (0.24)	15 (0.27)
Sickness absence days previous year	37 (0.33)	27 (0.48)
Employment status	0	0
SF-12 mental health	318 (2.83)	-
SF-36 vitality index	12 (0.11)	-
Body mass index	135 (1.20)	-
Diseases at baseline	0	-
Supervisory role in current position	31 (0.28)	-
Autonomy	14 (0.12)	-
Job demands	48 (0.43)	-
Emotional job demands	50 (0.45)	-
Financial situation in household	40 (0.36)	-
Current job is interesting	80 (0.71)	-
Independence at current job	86 (0.77)	-
Atmosphere at current job	86 (0.77)	-
Good salary at current job	85 (0.76)	-
Ability to change work hours at current job	83 (0.74)	-
Satisfaction with current job	87 (0.78)	-
Mastery skills	9 (0.08)	-
Values are n (%) unless stated otherwise. SF = Short Form Health Survey		

Online supplementary table 3. Loss to follow-up in the development and validation cohort

Characteristic	Level	**Development cohort** n=11,221			**Validation cohort** n=5,604		
		**Complete** n=8,681	**Missing at follow-up** n=2,540	**p-value**	**Complete** n=4,172	**Missing at follow-up** n=1,432	**p-value**
Male		4943 (56.9)	1459 (57.4)	0.65	2135 (51.2)	716 (50.0)	0.44
Age, mean (SD)		53.7 (5.2)	54.7 (5.8)	<0.001	50.2 (5.1)	50.4 (5.5)	0.25
Educational level[a]	Low	2216 (25.5)	708 (27.9)	0.02	979 (23.5)	411 (28.7)	<0.001
	Medium	3359 (38.7)	990 (39.0)		1685 (40.4)	568 (39.7)	
	High	3106 (35.8)	842 (33.1)		1508 (36.1)	453 (31.6)	
SF-12 physical health[b], mean (SD)		52.5 (6.9)	51.9 (7.4)	<0.001	52.2 (7.3)	52.0 (7.4)	0.48
Physically active[c]		3471 (40.3)	1031 (41.0)	0.53	1904 (45.8)	631 (44.4)	0.37
Physical job load[d], mean (SD)		1.8 (0.9)	1.8 (0.9)	0.11	1.9 (0.9)	1.9 (1.0)	0.55
Knowledge and skills match the job	Bad/mediocre	355 (4.1)	133 (5.3)	0.01	186 (4.5)	69 (4.9)	0.55
	Reasonable/good	8290 (95.9)	2397 (94.7)		3971 (95.5)	1352 (95.1)	
Major life events previous year	0	4580 (52.8)	1297 (51.1)	0.11	2426 (58.1)	813 (56.8)	0.64
	1	2766 (31.9)	811 (31.9)		1208 (29.0)	432 (30.2)	
	≥2	1335 (15.4)	432 (17.0)		538 (12.9)	187 (13.1)	
Work ability, mean (SD)		8.1 (1.3)	8.0 (1.4)	0.002	8.1 (1.4)	8.1 (1.5)	0.62
Sickness absence days previous year, mean (SD)		2.8 (5.0)	3.0 (5.2)	0.05	2.6 (4.8)	2.9 (5.2)	0.03
Occupational status	Employee	7788 (89.7)	2278 (89.7)	0.97	3773 (90.4)	1283 (89.6)	0.36
	Self-employed	893 (10.3)	262 (10.3)		399 (9.6)	149 (10.4)	

[a] Low: lower general secondary educational, preparatory secondary vocational education. Medium: intermediate vocational training, higher general secondary education, pre-university education. High: higher vocational education, university education.

[b] Weighted summary score (range 0-100) assessing physical health using 6 items of the 12-Item Short-Form Health Survey. Higher scores indicating better perceived physical health.

[c] Intensive physical exercise ≥3 days per week for ≥20 minutes.

[d] Average of five items (range: 1 = never, 5 = always) from the Dutch Musculoskeletal Questionnaire.(28)

Online Supplementary Table 4. Univariate associations and multivariable regression coefficients of the predictors in the final model of the development cohort in the complete cases (n=7,951)

		Univariate	Multivariable	
**Predictor**	**Level**	**OR (95%-CI)[a]**	**Odds ratio**	**Coefficient[b]**
Female gender		1.23 (1.01-1.48)	1.09	0.08
Age, per year		1.00 (0.99-1.02)	1.00	-0.003
Educational level[c] (ref: low)	Medium	0.99 (0.81-1.20)	0.85	-0.17
	High	0.74 (0.60-0.91)	0.81	-0.21
SF-12 physical health[d] (ref: 1st quartile, poorest health)	2nd quartile	1.02 (0.83-1.27)	0.53	-0.63
	3rd quartile	0.52 (0.41-0.66)	0.40	-0.91
	4th quartile	0.47 (0.36-0.61)	0.39	-0.94
Physically active [e]		0.73 (0.60-0.90)	0.77	-0.26
Physical job load[f] (ref: 1st-3rd quartile, less demanding)	4th quartile	1.63 (1.33-2.00)	1.32	0.28
Knowledge and skills match the job (ref: bad/mediocre)	Reasonable/good	0.45 (0.32-0.64)	0.65	-0.43
Major life events previous year (ref: none)	1	1.06 (0.87-1.30)	1.08	0.08
	≥2	1.58 (1.26-2.00)	1.39	0.33
Work ability[g] (ref: good)	Average	1.43 (1.16-1.76)	1.13	0.12
	Poor	4.76 (3.50-6.48)	2.27	0.82
Sickness absence days previous year (ref: none)	1 - 5	0.93 (0.75-1.17)	1.39	0.33
	6 - 10	2.25 (1.75-2.90)	2.50	0.92
	11 - 27	4.69 (3.71-5.92)	3.83	1.34
Self-employed		0.50 (0.33-0.75)	0.57	-0.55
Intercept				-2.01

[a] Odds Ratio (95% confidence interval); [b] Regression coefficients and intercept; an individuals predicted probability can be computed using the logistic regression formula P(LTSA) = 1/(1+exp(-LP), in which 'exp' denotes e-raised-to-the-power-of. The LP is the linear predictor, i.e. the linear sum of all predictor values multiplied by their regression coefficients, or -2.01 + 0.08*gender (female = 1) + 0.003*age (years) - 0.17*education (medium education = 1) -0.21*education (high education = 1) -0.63*physical health (2nd quartile = 1) -0.91*physical health (3rd quartile = 1) -0.94*physical health (4th quartile = 1) - 0.26*physically active (yes = 1) +0.28*physical job load (4th quartile = 1) -0.43*knowledge (reasonable/good = 1) +0.08*major life events (one event = 1) +0.33*major life events (two or more = 1) + 0.12*work ability (average = 1) +0.82*work ability (poor = 1) +0.33*sickness absence (1-5 days = 1) +0.92*sickness absence (6-10 days = 1) +1.34*sickness absence (11-27 days = 1) -0.55*employment status (self-employed = 1)

[c] Low: lower general secondary educational, preparatory secondary vocational education. Medium: intermediate vocational training, higher general secondary education, pre-university education. High: higher vocational education, university education.

[d] Weighted summary score (range 0-100) assessing physical health using 6 items of the 12-Item Short-Form Health Survey. Higher scores indicating better perceived health. 1st quartile < 46.1 , 2nd quartile = 46.1 - 54.1, 3rd quartile = 54.2 – 56.5, 4th quartile >= 56.6.

[e] Intensive physical exercise ≥3 days per week for ≥20 minutes.

[f] Average of five items (range: 1 = never – 5 = always) from the Dutch Musculoskeletal Questionnaire.(28) 1st-3rd quartile <2.4 , 4th quartile >= 2.4.[g] Measured with the first item of the Work Ability Index (WAI).(29) Good = 8-10, average = 6/7, poor = 0-5

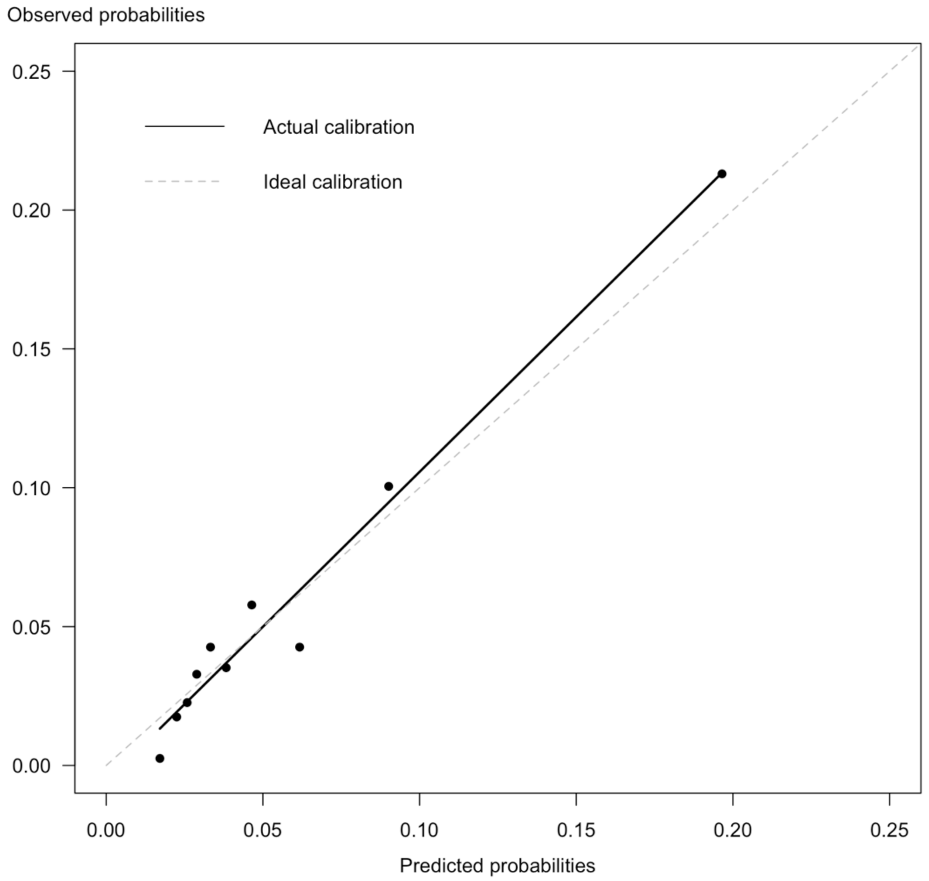

Online Supplementary Figure 1. Calibration plot visualizing the mean predicted LTSA-risk by the model against observed frequencies per decile of predicted risk in the complete cases (n = 7,951) of the validation cohort. Hosmer-Lemeshow test: p=1.00
